# Middle East Respiratory Syndrome-Coronavirus Infection into Established hDPP4-Transgenic Mice Accelerates Lung Damage Via Activation of the Pro-Inflammatory Response and Pulmonary Fibrosis

**DOI:** 10.4014/jmb.1910.10055

**Published:** 2019-12-15

**Authors:** Ju Kim, Ye Lin Yang, Yongsu Jeong, Yong-Suk Jang

**Affiliations:** 1 Department of Molecular Biology and the Institute for Molecular Biology and Genetics, Jeonbuk National University, Jeonju 54896, Republic of Korea; 2 Department of Bioactive Material Sciences and Research Center of Bioactive Materials, Jeonbuk National University, Jeonju 54896, Republic of Korea; 3 Graduate School of Biotechnology, Kyung Hee University, Yongin 17104, Republic of Korea

**Keywords:** Animal model, hDPP4, MERS-CoV, pathogenesis, transgenic mouse

## Abstract

Middle East respiratory syndrome coronavirus (MERS-CoV) infects the lower respiratory airway of humans, leading to severe acute respiratory failure. Unlike human dipeptidyl peptidase 4 (hDPP4), a receptor for MERS-CoV, mouse DPP4 (mDPP4) failed to support MERS-CoV infection. Consequently, diverse transgenic mouse models expressing hDPP4 have been developed using diverse methods, although some models show no mortality and/or only transient and mild-to-moderate clinical signs following MERS-CoV infection. Additionally, overexpressed hDPP4 is associated with neurological complications and breeding difficulties in some transgenic mice, resulting in impeding further studies. Here, we generated stable hDPP4-transgenic mice that were sufficiently susceptible to MERS-CoV infection. The transgenic mice showed weight loss, decreased pulmonary function, and increased mortality with minimal perturbation of overexpressed hDPP4 after MERS-CoV infection. In addition, we observed histopathological signs indicative of progressive pulmonary fibrosis, including thickened alveolar septa, infiltration of inflammatory monocytes, and macrophage polarization as well as elevated expression of profibrotic molecules and acute inflammatory response in the lung of MERS-CoV-infected hDPP4-transgenic mice. Collectively, we suggest that this hDPP4-transgenic mouse is useful in understanding the pathogenesis of MERS-CoV infection and for antiviral research and vaccine development against the virus.

## Introduction

Middle East respiratory syndrome-coronavirus (MERS-CoV) infects the lower respiratory airway of humans and leads to acute respiratory distress syndrome with approxi-mately 34.5% mortality [1, 2]. Symptoms caused by MERS-CoV infection are similar to those of severe acute respiratory syndrome-coronavirus (SARS-CoV) infection, but, unlike SARS-CoV, MERS-CoV uses the species-specific receptor, human dipeptidyl peptidase 4 (hDPP4), for its entry into host cells [3, 4]. In humans, hDPP4 is continuously expressed in epithelial cells and activated leukocytes [5], and MERS-CoV infects human tracheobronchial epithelium, bronchiolar epithelial cells, alveolar adenocarcinoma, and liver cell line, such as Huh-7 [6]. Small animals such as the mouse [7], ferret [8], and hamster [9] are non-permissive to MERS-CoV [10, 11]. Especially, structural differences between mouse DPP4 and hDPP4 at the interface with the receptor-binding domain of the MERS-CoV spike protein are reported to influence susceptibility to MERS-CoV infection [12]. Nonhuman primates such as rhesus macaques and common marmosets are sensitive to MERS-CoV infection, but they exhibit only transient and mild clinical symptoms with occasional fatal interstitial pneumonia following MERS-CoV infection [13, 14]. Furthermore, application of nonhuman primates for studies on the pathogenesis of MERS-CoV infection and evaluation of antiviral agents and vaccines is limited due to ethical and cost-effectiveness aspects, and the need for sophisticated operations. Consequently, since the isolation and identi-fication of MERS-CoV in 2012, research has focused on developing appropriate animal models.

Development of mouse models for MERS-CoV infection and the infection-induced disease has been attempted using constitutive, inducible, and tissue-specific promoters. The first MERS-CoV infectious mouse model was generated using recombinant non-replicating adenovirus expressing hDPP4. However, the transgenic mice exhibited no mortality and showed only progressive interstitial pneumonia following MERS-CoV infection [15]. Although additional transgenic mouse models expressing hDPP4 were established, they revealed complicated morbidity with neurological complications, high mortality correlated with brain damage, and breeding difficulty following MERS-CoV infection due to overexpressed DPP4 [16, 17]. In addition to functioning as a MERS-CoV receptor, DPP4 has diverse biological functions, including regulation of cytokines and incretin hormones, and T cell activation/migration to inflammatory sites [18-20]. Besides, pulmonary viral infection often induces a hyperinflammatory response [21] and leads to fibrosis due to uncontrolled sequential phases of infla-mmation, injury, and tissue repair [22]. During continuous fibrosis, lung tissue is gradually destroyed, resulting in a loss of organ function instead of extracellular matrix (ECM) reorganization [23]. These outcomes are thought to occur during a dysregulated early inflammatory response and exacerbate the morbidity and mortality of the transgenic mice.

In this study, we generated transgenic mice expressing hDPP4 (hDPP4-Tg) through microinjection of a pTK-hDPP4 gene construct into a fertilized egg from the FVB/N mouse strain, because the mice facilitate transduction of foreign genes due to the presence of large pronuclei, a high rate of pronuclear fusion, and a predominant litter size. The transgenic founder mice were identified and crossed with nontransgenic C57BL/6 mice to propagate hDPP4-Tg mice, retaining the germline transmission ability in the C57BL/6 background. Following intranasal administration of MERS-CoV to hDPP4-Tg mice, we confirmed mortality and morbidity with inflammatory response and lung damage, which are closely associated with the clinical signs. Histopathological analyses ascertained the degree of lung damage accompanied by progressive fibrosis and changes of expressed profibrotic molecules in the lungs of MERS-CoV-infected hDPP4-Tg mice.

## Materials and Methods

### Experimental Animals and Materials

Six- to eight-week-old female FVB/N and C57BL/6 mice were purchased from the Koatech Laboratory Animal Center (Pyeongtaek, Korea) and housed under specific pathogen-free conditions with water and food provided *ad libitum*. Animal experiments were approved by the Institutional Animal Care and Use Committee of Jeonbuk National University (Approval No. CBNU 2017-0056) and followed the guidelines suggested by the committee. Vero E6 (ATCC CRL-1586) cells were obtained from the American Type Culture Collection (Manassas, USA). MERS-CoV (1-001-MER-IS-2015001) was obtained from the Korea Centers for Disease Control and Prevention. All experiments using MERS-CoV were performed in accordance with the World Health Organization recommendations in a biosafety level 3 facility in the Korea Zoonosis Research Institute at Jeonbuk National University (Korea). Unless otherwise specified, the chemicals and laboratory equipment used in this study were obtained from Sigma Chemical Co. (USA) and SPL Life Sciences (Korea), respectively.

### Determination of hDPP4-Expressing Transgenic Mice

The transgene comprising the thymidine kinase (TK) promoter, full-length DNA of hDPP4 with the N-terminal Flag tag, and the poly (A) signal was released by plasmid cloning the pTK-hDPP4 gene construct using the NotI restriction enzyme and microinjected into a fertilized egg from the FVB/N mouse strain. Injected zygotes were subsequently implanted into pseudopregnant recipients, and the resulting offspring were genotyped for hDPP4 transgene integration using genomic DNA isolated from tail biopsies. The hDPP4-specific primers for genotyping were as follows: forward, 5’-GGCCTCGAACACCGAGCGAC-3’ and reverse, 5’-GATGCT ACAGCTGACAGTCG-3’. Nine genotype-positive transgenic founders were back-crossed with C57BL/6 mice to produce the F1 generation and to confirm germline transgenicity. Except founder line 4, which was incapable of germline transmission, tail tips of hDPP4-positive F1 mice from eight identified fertile founders of the transgenic lineage were subjected to western blotting to confirm stable expression of the hDPP4 protein. Briefly, tail tips from identified hDPP4-Tg mice were homogenized in RIPA lysis buffer (50 mM Tris-HCl, pH 7.4, containing 150 mM NaCl, 100 mM EDTA, and 0.1% SDS) supplemented with a complete protease inhibitor cocktail (Roche Applied Science, Germany). The denatured protein lysates were prepared by centrifugation at 10,000 ×*g* at 4°C for 10 min. Equal amounts of lysates were resolved by sodium dodecyl sulfate-polyacrylamide gel electrophoresis (SDS-PAGE), transferred to an Immobilon-P polyvinylidene difluoride membrane (Merck Millipore, USA), and immunoblotted using specific antibodies (Abs). The primary Ab against Flag tag and the Ab against mouse β-actin were purchased from Sigma Chemical Co.(USA). Target proteins were detected by enhanced chemilumi-nescence (Thermo Fisher Scientific, USA).

Among four transgenic lineages showing distinct expression of hDPP4, founder line 27 was highly fertile and gave birth to large litters with healthy pups. So, we tested for the distribution of hDPP4 expression in various tissues of founder line 27, including brain, lung, liver, and intestine using western blotting according to the above-described procedure. This identified transgenic lineage was continuously crossed with C57BL/6 mice to generate many hDPP4-Tg offspring that provided additional animals for further biological and pathological analyses of the outcome of MERS-CoV challenge infection.

### Viral Challenge and Sample Collection

MERS-CoV was propagated in Vero E6 cells, which were grown in Dulbecco’s Modified Eagle’s Medium (Welgene, Korea) supplemented with 10% FBS (Gibco-Thermo Fisher Scientific) at 37°C in a humidified CO_2_ incubator. MERS-CoV was passed six times in Vero E6 cells and used to assess the morbidity and mortality in the identified hDPP4-Tg mice. Briefly, hDPP4-Tg and their transgene-negative littermates were anesthetized and inoculated intranasally with 105 plaque-forming units (PFUs) of MERS-CoV in a total volume of 60 μl. hDPP4-Tg and transgene-negative littermates were weighed and monitored daily for clinical signs of disease, including appearance, abnormalities of behavior or movements, decreased activity or enhanced responsiveness, weight loss, and death. Some infected mice were sacrificed at the indicated time points post-infection to obtain tissue specimens to assess the viral loads and the expression levels of target genes associated with the histopathological changes using real-time quantitative reverse transcription-polymerase chain reaction (qRT-PCR) and histopathologic evaluation by hematoxylin-eosin (H&E) staining.

### RNA Extraction and qRT-PCR

Tissue specimens, including lung, brain, liver, spleen, and intestine from MERS-CoV-infected mice were weighed and transferred to individual vials containing TRIzol reagent (Thermo Fisher Scientific). The collected tissues were homogenized and subsequently subjected to total RNA isolation as previously described [24]. RNA was extracted using TRIzol reagent according to the manufacturer’s instructions. RNA was converted into cDNA using an MMLV Reverse Transcription Kit (Promega, Fitchburg, USA). Gene expression was quantified by qRT-PCR with the SsoAdvanced Universal SYBR Green Supermix (Bio-Rad Laboratories, USA) and a CFX Connect Real-Time system (Bio-Rad Laboratories) using 50 ng of first-strand cDNA under the following conditions: 95°C for 5 min followed by 40 cycles at 95°C for 15 sec, 55°C for 30 sec, and 72°C for 30 sec. The relative expression level of each gene was obtained by normalizing with the endogenous control gene, β-actin, and was calculated in terms of the fold difference using CFX Maestro software (Bio-Rad Laboratories). The primers used are listed in Table 1.

### Histopathology

Tissues obtained from MERS-CoV- and sham-infected mice at the indicated time points were immediately fixed in 10% neutral buffered formalin, transferred to 70% ethanol, and paraffin embedded. Histopathologic evaluation was performed on tissue sections deparaffinized and stained by H&E. Pathological signs such as denatured and collapsed cell/tissue organization, hemorrhage in the interstitial space, infiltration of inflammatory monocytes, and change of alveolar septa after MERS-CoV infection were examined.

### Statistical Analysis

Statistical analyses were performed using Prism 7 (GraphPad, USA). Data are presented as means ± standard deviations. The statistical significance of numerical data was analyzed using a two-way analysis of variance (ANOVA), and *p* < 0.05 was considered statistically significant.

## Results

### Establishment of Transgenic Mice Expressing hDPP4

**Fig. 1:**
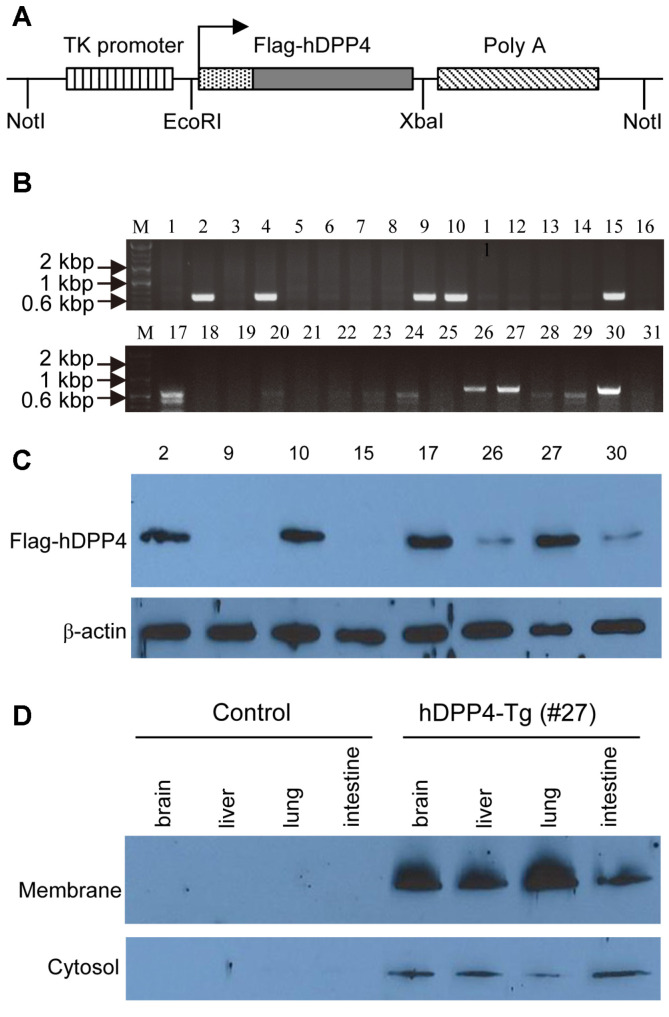
Generation and characterization of hDPP4 transgenic (hDPP4-Tg) mice. (**A**) Schematic diagram of the gene construct for constitutive expression of hDPP4 in transgenic mice. The Flag tag fused hDPP4 (Flag-hDPP4) was cloned into a plasmid (pTK-hDPP4) in which hDPP4 expression was driven by the TK promoter. (**B**) Determination of genotypes in 31 hDPP4 transgenic founders by hDPP4-specific primers. Genomic DNA extracted from tail biopsies of hDPP4 transgenic founders capable of germline transmission were subjected to PCR analyses to determine the integrated hDPP4 transgene, as described in the Materials and Methods. (**C**) Confirmation of hDPP4 expression in eight transgenic founder lineages by western blot analyses. Protein lysates extracted from tail tips of eight genotypepositive transgenic founder mice were subjected to western blot analyses to determine the relative expression of hDPP4 protein, as described in the Materials and Methods. (**D**) Expression of the hDPP4 transgene in the indicated tissues of transgenic founder line 27 as determined by western blot analyses. Various tissue specimens of hDPP4-Tg mice derived from lines 27 and the transgene-negative littermate control mice were homogenized to extract protein lysates before the expression of hDPP4 protein was assessed by western blotting with the hDPP4-specific antibody described in the Materials and Methods.

DPP4 is expressed in the parenchyma cells of various tissues, including T and B cells in humans [5]. We generated pTK-hDPP4 where hDPP4 gene expression was controlled by the TK promoter, and we used the gene construct to generate transgenic mice (Fig. 1A). The pTK-hDDP4 expression cassette was linearized by NotI digestion and microinjected into the pronuclei of fertilized eggs from FVB/N mice. Injected zygotes were subsequently implanted into pseudopregnant recipients, which led to 31 healthy offspring. Transgenic founders able to transfer the hDPP4 gene to their progeny were determined by PCR using genomic DNA prepared from tail biopsies (Fig. 1B). We selected nine transgenic founders based on the genotyping results and crossed them with C57BL/6 mice to propagate the lines. Transgenic founder line 4 appeared to be incapable of transmitting the transgene due to a failure to give birth. Besides, hDPP4-Tg founder lines 2, 10, 17, and 27 were identified as being able to transmit the hDPP4 transgene to their offspring and showed stable expression of the hDPP4 transgene. Western blot analyses revealed the highest intensity of hDPP4 expression to be in line 27, followed by lines 17, 10, and 2 in descending order (Fig. 1C). Importantly, lines 2, 10, and 17 had small litters and poor growth of pups, whereas line 27 exhibited high fertility and produced large litters with healthy pups.

We analyzed the expression and tissue distribution of hDPP4 in line 27 by western blotting with proteins prepared from brain, liver, lung, and intestine (Fig. 1D). Compared to the results from the transgene-negative littermate control mouse, hDPP4 expression was present in all the tissues tested in line 27 and the expression level of hDPP4 was higher in the lung than in other tested tissues. In addition, the founder line 27 was capable of producing first-, second-, and third-generation hDPP4-Tg pups, which survived to maturity and expressed the hDPP4 transgene. Taken together, we speculated that transgenic founder line 27 is a stable transgenic lineage globally expressing hDPP4, a receptor for MERS-CoV infection.

### hDPP4-Tg Mice Are Permissive to MERS-CoV Infection that Results in Increased Morbidity and Mortality and Proinflammatory Cytokine and Chemokine Expression

**Fig. 2:**
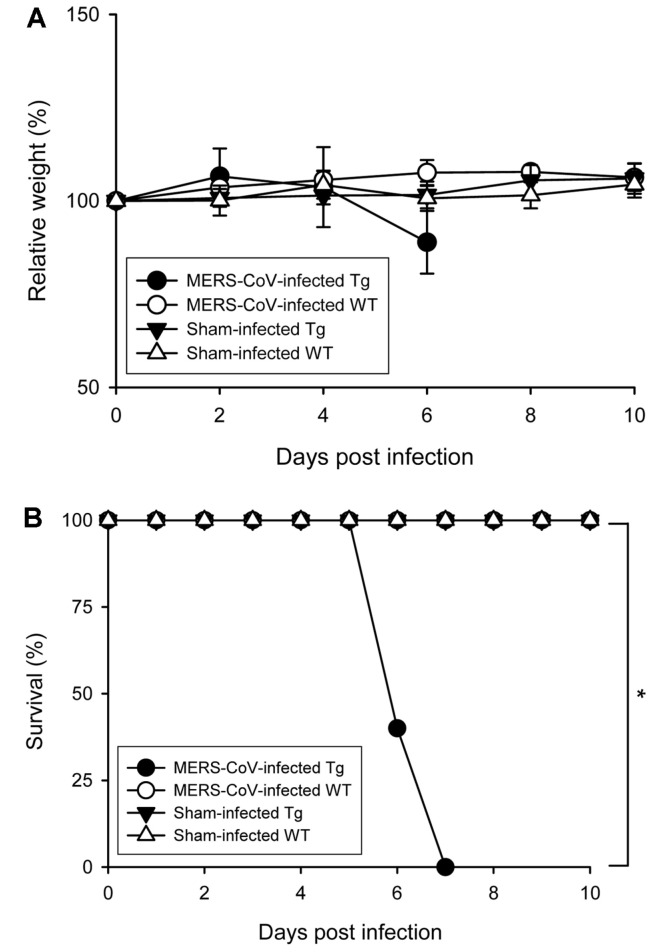
Human DPP4-Tg mice are permissive to MERS-CoV infection that results in increased morbidity and mortality. Both hDPP4-Tg mice and transgene-negative littermates (WT) were challenged intranasally with 10^5^ PFU of MERS-CoV or PBS in 60 μl. Infected mice were monitored daily for weight changes, other signs of clinical illness, and mortality. (**A**) Bodyweight changes of MERSCoV- and sham-infected (60 μl of PBS) mice. Results are expressed as means ± standard deviation (SD; *n* = 5) at the indicated times postinfection. (**B**) Survival curves of hDPP4-Tg mice and transgenenegative littermates (WT) after MERS-CoV and sham infection (*n* = 5). *p < 0.05.

Wild-type mice, including BALB/c, C57BL/6, and FVB/N are not permissive to MERS-CoV infection and do not support MERS-CoV replication [8-11]. To characterize the established hDPP4-Tg in terms of MERS-CoV infection, we inoculated 105 PFUs of MERS-CoV intranasally into hDPP4-Tg and age-matched, transgene-negative littermates. The mice were monitored daily for clinical signs, including weight loss and mortality (Fig. 2). Only the hDPP4-Tg mice developed acute respiratory and wasting syndromes with rapid weight loss at 4 days post-infection (dpi) to 6 dpi, and subsequently 40% and 100% mortality at 6 dpi and 7 dpi, respectively. Other clinical symptoms, including ruffled or sweaty fur, inactivity, convulsions, and more rapid breathing, were also observed in MERS-CoV-infected hDDP4-Tg mice.

**Fig. 3:**
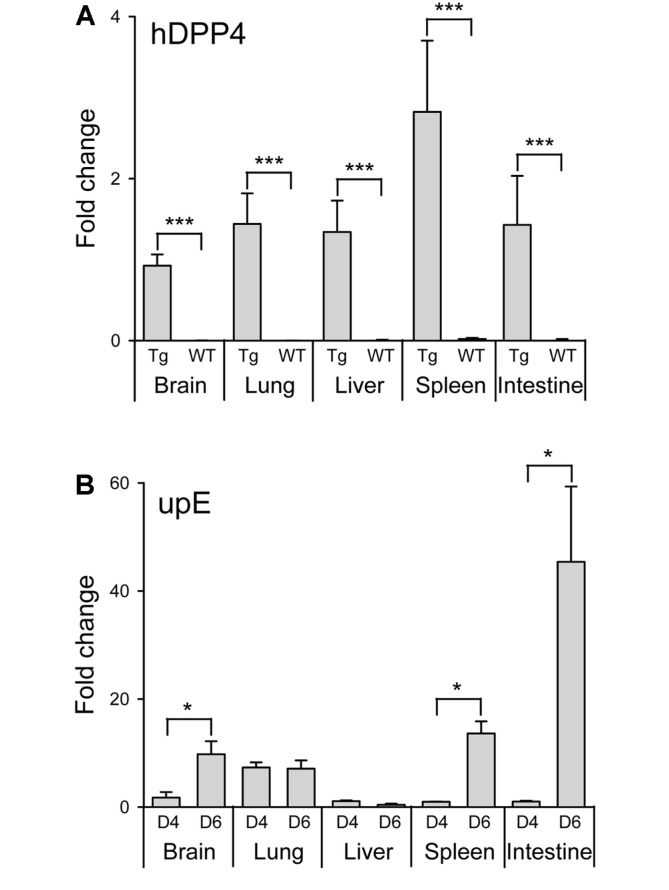
Determination of hDPP4 transgene expression and viral load in the tissues of hDPP4-Tg mice infected with MERS-CoV. (**A**) Indicated tissues of hDPP4-Tg mice (Tg) and transgene-negative littermates (WT) were homogenized to extract total RNA to assess the relative abundances of hDPP4 expression by real-time quantitative RT-PCR (qRT-PCR) of the hDPP4 gene with normalization to that of the internal control gene, β-actin. (**B**) Viral loads in the indicated tissues of MERS-CoV-infected hDPP4-Tg mice on days 4 (D4) and 6 (D6) post-infection were determined by qRT-PCR of the viral RNA targeting the upstream E (upE) gene with normalization to that of the internal control gene, β-actin. Results are expressed as means ± SD. *p < 0.05 and ***p < 0.001.

To determine the tissue distribution of the hDPP4 transgene and viral gene expression following MERS-CoV infection, tissue specimens of hDPP4-Tg and transgene-negative littermates collected 4 and 6 dpi were analyzed by qRT-PCR using hDPP4 and MERS-CoV upstream E (upE) gene-specific primer sets (Fig. 3). The results showed that disseminated infection of MERS-CoV was present only in hDPP4-Tg mice, but not in the transgene-negative littermates. The levels of viral RNA in brain, spleen, and intestine increased significantly in a time-dependent manner, as evidenced by the increase of viral upE gene expression at 6 dpi (Fig. 3B). However, there was only low or unchanged viral upE gene expression in the liver, despite expression of hDPP4 in all the tissues tested (Fig. 3A).

**Fig. 4:**
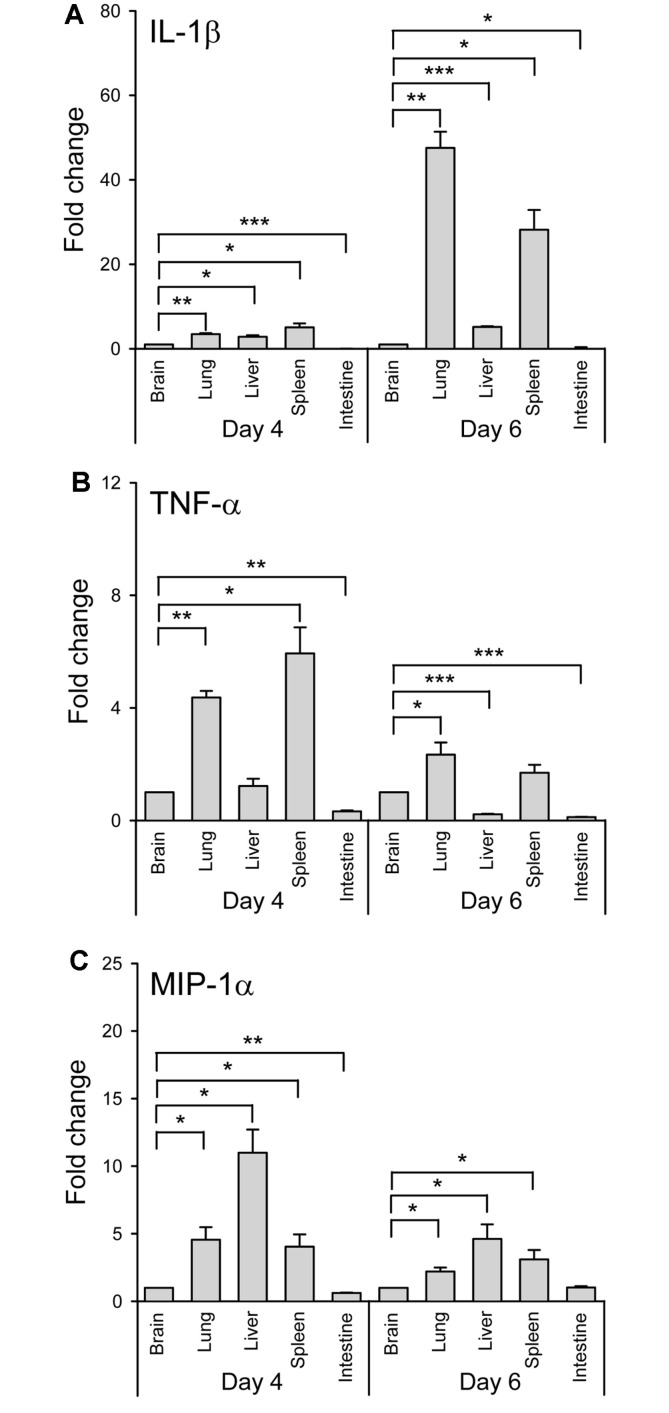
Identification of acute innate inflammatory responses in the tissues of hDPP4-Tg mice infected with MERS-CoV. Total RNA was extracted from the indicated tissues of hDPP4-Tg mice on days 4 and 6 after infection with MERS-CoV and processed to assess the innate inflammatory responses by qRT-PCR. The relative mRNA expression of IL-1β (**A**), TNF-α (**B**), and MIP-1α (**C**) was analyzed by qRT-PCR in duplicate, with normalization to the expression of the internal control gene, β-actin. Results are expressed as means ± SD. *p < 0.05, **p < 0.01, and ***p < 0.001.

Next, we analyzed indicators of the inflammatory response in the tissues of hDPP4-Tg mice following MERS-CoV infection, because changes in cytokine and chemokine responses in patients severely infected with MERS-CoV are important aspects responsible for MERS-CoV pathogenesis (Fig. 4) [14,15,25-27]. Specifically, using qRT-PCR, we determined the mRNA expression levels of the representative proinflammatory cytokines, IL-1β and TNF-α, as well as the chemokine, MIP-1α, which were selected based on previous in vivo studies of respiratory viral infections, including SARS-CoV [28, 29]. Elevated expression of both IL-1β and TNF-α was prominently observed in the lung and spleen at 4 and 6 dpi (Figs. 4A and 4B), while elevated expression of MIP-1α was prominently observed in the liver, lung, and spleen (Fig. 4C). These results suggest that hDPP4-Tg mice support MERS-CoV infection, resulting in morbidity and mortality; they also rapidly elicit acute local and subsequent systemic inflammatory responses following MERS-CoV infection.

### MERS-CoV Infection of hDPP4-Tg Mice Induced Histopathological Changes in the Lung

**Fig. 5:**
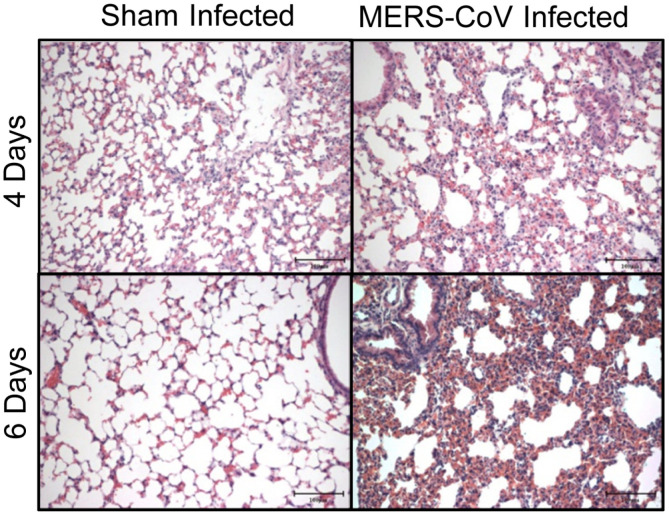
Histopathological changes in the lungs of hDPP4-Tg mice challenged with MERS-CoV. hDPP4-Tg mice were infected intranasally with MERS-CoV. Lung tissues were collected 4 and 6 days after MERS-CoV infection, fixed, and paraffin-embedded sections of lung specimens were stained with hematoxylin and eosin. Histopathological analyses were performed using shaminfected (60 μl of PBS) hDPP4-Tg mice as controls (scale bars = 100 μm).

As the lungs represent the initial site of respiratory virus infection, histopathological analysis of lung tissues was conducted at 4 and 6 dpi (Fig. 5). Compared to the lung tissues from sham-infected mice, time-dependent progressive lung damage was observed in MERS-CoV-infected hDPP4-Tg mice. At 6 dpi, hDPP4-Tg mice exhibited irregular arrangement of pneumocytes, alveolar septal changes, and mild inflammation with inflammatory cell infiltration into the lung. In addition, the lung damage became severe with progressive pulmonary fibrosis, including alveolar septal thickening, focal hemorrhage in the interstitium, and activated macrophage infiltration at 6 dpi. However, no histopathological changes were detected in lung tissues prepared from sham-infected hDPP4-Tg mice.

**Fig. 6:**
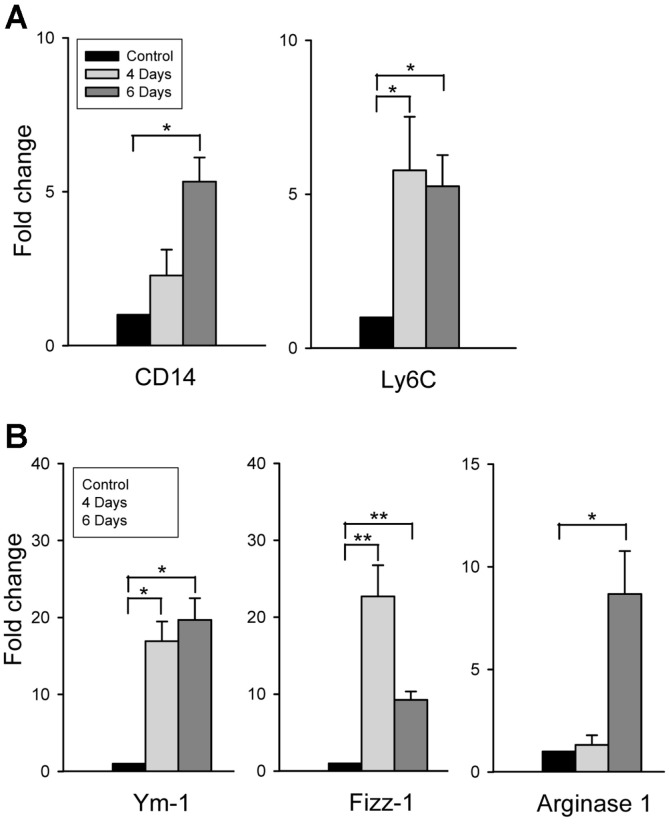
Detection of polarized activation of macrophages in the lungs of hDPP4-Tg mice infected with MERS-CoV. Total RNA was extracted from lung tissues of hDPP4-Tg mice 4 and 6 days after infection with MERS-CoV and processed to evaluate changes in pulmonary macrophage polarization status by qRT-PCR. The relative expression of genes for (**A**) macrophage activation and (**B**) M2 surface markers, together with that of the internal control gene, β-actin, was analyzed by qRT-PCR in duplicate. Expression levels relative to those of the non-treated controls are shown as means ± SD. *p < 0.05 and **p < 0.01.

The aberrant immune response is assumed to be related to the pathogenesis of MERS-CoV infection and the related clinical symptoms. Given that a systemic inflammatory response was enhanced by MERS-CoV infection in hDPP4-Tg mice, which was confirmed by a significant increase in proinflammatory cytokine and chemokine expression in the spleen, and that macrophage infiltration was increased in the lungs of MERS-CoV-infected hDPP4-Tg, we assume that macrophage activation is related with an aberrant inflammatory response in MERS-CoV-infected hDPP4-Tg mice. To clarify this, we analyzed the factors involved in macrophage activation (Fig. 6). Expression of CD14, whose expression is increased by macrophage activation, was significantly (*p* < 0.05) increased in hDPP4-Tg at 6 dpi compared to that of the control. Expression of Ly6C, an inflammatory monocyte marker abundantly expressed on the surface of monocytes, was also significantly (*p* < 0.05) increased in hDPP4-Tg at 4 and 6 dpi compared to that of the control (Fig. 6A). Next, we investigated if the increased inflammatory response influenced macrophage polarization, and thus, the progress of pulmonary fibrosis. Expression of the M2 markers Ym-1 and Fizz-1 was significantly enhanced at 4 and 6 dpi in hDPP4-Tg mice compared to that of the control, while expression of another M2 marker, Arginase1, was significantly enhanced at 6 dpi in hDPP4-Tg mice compared to that of the control (Fig. 6B). By contrast, there was only a slight increase in the expression of the M1 marker genes (CD16 and CD80) at 4 dpi, and, overall, no significant difference in M1 marker gene expression between MERS-CoV-infected and non-treated control hDPP4-Tg mice (data not shown). Collectively, these results indicate that the expression of Ym-1, Fizz-1, and Arginase1 was predominant in the lungs of hDPP4-Tg mice following infection with MERS-CoV, suggesting M2 macrophage polarization.

### MERS-CoV Infection in hDPP4-Tg Mice Promoted Pulmonary Fibrosis

**Fig. 7:**
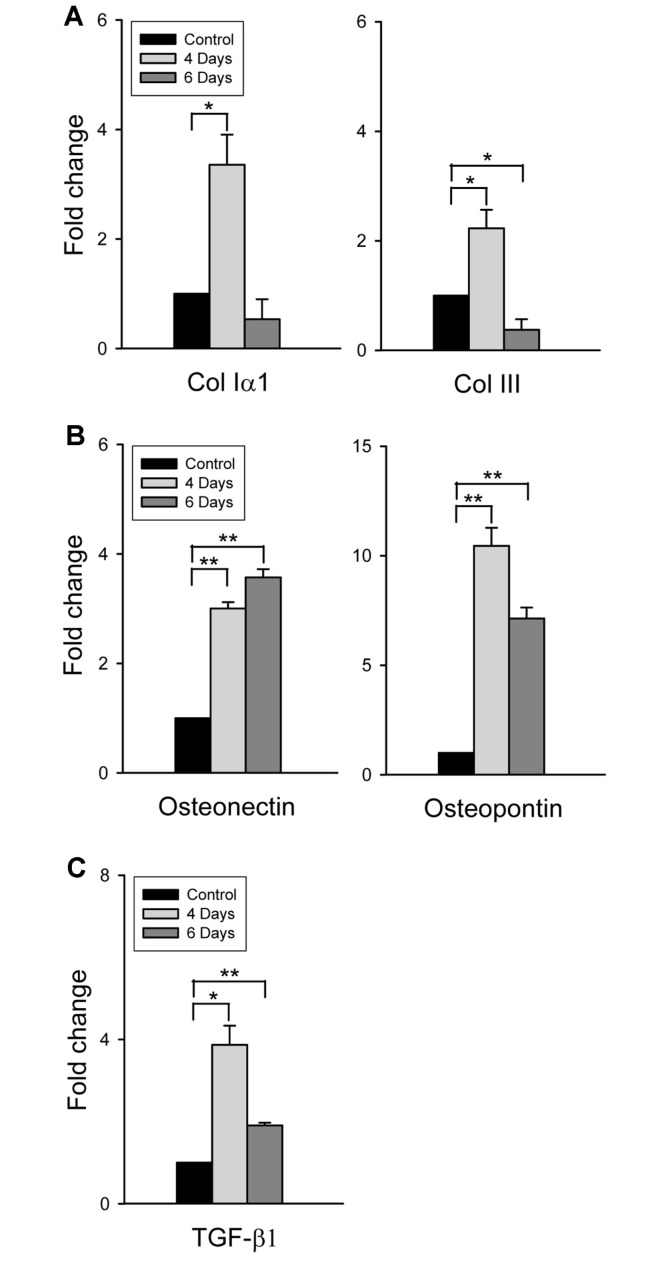
Identification of progressive pulmonary fibrosis in the tissues of hDPP4-Tg mice infected with MERS-CoV. Total RNA was extracted from the lung tissues of hDPP4-Tg mice 4 and 6 days after infection with MERS-CoV and processed to estimate pulmonary fibrosis by qRT-PCR. The relative abundance of profibrotic molecules and cytokine gene expression, together with that of the internal control gene, β-actin, was analyzed by qRT-PCR in duplicate. Expression levels relative to those of the non-treated controls are shown as means ± SD. *p < 0.05 and **p < 0.01.

The production and deposition of ECM components such as collagen are representative features of pulmonary fibrosis [30]. The common structural collagens are composed of collagen type I (Col Iα1 and Iα2) and collagen type III (Col IIIα1). MERS-CoV infection in hDPP4-Tg mice promoted the expression of Col Iα1 (*p* < 0.05) and Col III in lung tissues at 4 dpi compared to the control group, but the increasing effect was abrogated over time (Fig. 7A). Furthermore, expression of osteonectin and osteopontin, which have profibrotic effects in the development of pulmonary fibrosis [31, 32], was also promoted significantly (*p* < 0.01) and was maintained in the lung tissues of MERS-CoV-infected hDPP4-Tg mice compared to those in the control group (Fig. 7B). Osteonectin and osteopontin also have a profibrotic effect in the development of pulmonary fibrosis. A notable morphological feature of pulmonary fibrosis is the development of fibroblastic foci, representing active fibrogenesis. Osteonectin is accumulated in myofibroblasts at fibroblastic foci and, locally, stimulates collagen deposition, recruitment of inflammatory cells, TGF-β1 production, and synthesis of ECM proteins during the lung fibrogenesis. Osteopontin is also a key proin-flammatory cytokine involved in tissue repair. Osteopontin induces the growth and migration of fibroblasts, and promotes ECM deposition via TGF-β1 activation and type I collagen expression. Given that the expression of diverse profibrotic cytokines is the hallmark of fibrosis and the most prominent factor known to promote the progression of pulmonary fibrosis is TGF-β1 [33], we analyzed the expression of TGF-β1 in the lung tissues of MERS-CoV-infected mice and compared it with that in non-treated control hDPP4-Tg mice. Consistent with previous findings of collagen synthesis and histological changes, we observed significantly enhanced TGF-β1 expression which peaked at 4 dpi (*p* < 0.05), and decreased at 6 dpi (*p* < 0.01) in the lung tissues of MERS-CoV-infected hDPP4-Tg mice (Fig. 7C). These results indicate that lung damage is aggravated by enhanced inflammatory and fibrosis responses resulting from MERS-CoV infection in hDPP4-Tg mice. Moreover, lung damage is believed to be the primary reason underlying the severe clinical outcome in hDPP4-Tg mice following MERS-CoV infection.

## Discussion

The lack of a reliable animal model system has hampered research of the pathological mechanisms caused by MERS-CoV infection and hence the development of vaccines and antiviral agents against MERS-CoV. To develop a reliable model susceptible to MERS-CoV, stable transgenic mouse models expressing hDPP4 or mouse DPP4 edited through the CRISPR/Cas9 system to accommodate MERS-CoV infection were generated and, after which progressive pneumonia and lethal effects in the transgenic mice were confirmed following MERS-CoV infection [34, 35]. Although these transgenic mice suffice for evaluation of the efficacy of vaccines or antiviral agents, their use remains limited due to breeding difficulties and the confounding morbidity with neurological complications, such as brain damage, rather than pulmonary failure, resulting from MERS-CoV infection [36]. Vaccines and immunotherapeutics have not been able to provide protection against brain damage due to their inability to cross the blood-brain barrier and are thus of limited use even if they display protective and/or therapeutic efficacy in the lung [37]. Moreover, given that respiratory infection and pathological damage in the lungs are key aspects in severe acute human disease caused by MERS-CoV infection, a transgenic mouse model with minimal confounding effects resulting from overexpressed DPP4 as well as pathology in extra-pulmonary tissues is needed to determine their impacts on MERS-CoV-induced disease.

In this study, we generated transgenic mice globally expressing hDPP4 by microinjecting the pTK-hDPP4 gene construct into a fertilized egg prepared from the FVB/N mouse strain. The FVB/N mice are feasible for transducing foreign genes due to the large pronuclei, the high rate of pronuclear fusion, and the predominant litter size. According to previous studies, an increased DPP4 expression has been reported to negatively affect the growth capacity of endothelial cells within the microvasculature network [38]. And it is speculated that glucose dysmetabolism and abnormal vasculature in some hDPP4 transgenic mice are responsible for breeding difficulty and prenatal or neonatal death of pups [17]. Consequently, we identified transgenic founder mice with high fertility that produced healthy offspring (n > 50), which were crossed with nontransgenic C57BL/6 mice to stably propagate hDPP4-Tg mice that maintained the germline transmission ability with a C57BL/6 background. hDDP4-Tg mice exhibited several defined endpoint signs, including rapid weight loss and death following MERS-CoV challenge infection (Fig. 2). Enhanced expression of acute and chronic cytokines, such as IL-1β, TNF-α, and MIP-1α was also detected, especially in the lung (the initial infection site), and spleen (a representative tissue of systemic immunity) of the transgenic mice following challenge with MERS-CoV (Fig. 4). Additionally, we observed that most of the hDDP4-Tg mice that died within days following MERS-CoV challenge had developed severe respiratory dysfunction with pathological changes in the lung. Interestingly, some of the transgenic mice exhibited additional symptoms, such as imbalance and gait difficulty. However, conspicuous pathological damage was not observed in brain tissues from MERS-CoV-challenged transgenic mice, even when a high level of viral RNAs was detected in the brain tissues following MERS-CoV infection (data not shown). Consequently, further analysis is needed to clarify whether the neurological manifestation is due to the direct effect of virus replication or aberrant inflammatory responses promoted by virus replication. Interestingly, increased levels of viral RNAs were observed in the spleen and intestines of MERS-CoV-infected hDPP4-Tg mice at 6 dpi (Fig. 3B), although no noticeable histopathological changes were detected in these tissues. In addition, a very low level of proinflammatory cytokines and chemokines was detected in the intestines of MERS-CoV-infected hDPP4-Tg mice. Innate immune responses on mucosal surfaces of the gastrointestinal tract following viral infection are reported to be different depending on the virus type [39, 40]. Specifically, compared to rotavirus, coronavirus did not induce a detectable proinflammatory response, despite the activation of p50 of NF-κB in early virus-infected intestinal loops. It is also important to note that TNF, a multi-functional proinflammatory cytokine, was repressed by both rotavirus and coronavirus, but IRF1, which is required to activate type I IFN responses, was activated following rotavirus infection. These differences in innate immune responses indicate that viruses have different strategies to evade host defense mechanisms. Collectively, the delayed proinflammatory response at early coronavirus infection is believed to result in a prolonged infection. These results suggest that MERS-CoV infection was predominant in the respiratory tract, and especially within the lung, leading to dissemination of the virus to other tissues. Therefore, lung infection and pathological damage in the lung were the most critical causative factors for morbidity and mortality caused by MERS-CoV infection in the hDPP4-Tg mice.

Besides promoting inflammation, acute viral infection also promoted fibrosis of infected tissue from the start of the infection. A notable morphological feature of pulmonary fibrosis is the development of fibroblastic foci representing active fibrogenesis [41]. Osteonectin accumulates in myofibroblasts at fibroblastic foci and, locally, osteonectin stimulates collagen deposition, recruitment of inflammatory cells, TGF-β1 production, and synthesis of ECM proteins during lung fibrogenesis. Osteopontin is also a key proinflammatory cytokine involved in tissue repair; it induces growth and migration of fibroblasts and promotes ECM deposition through the expression of TGF-β1 and type I collagen. Moreover, TGF-β1 is the most prominent profibrotic cytokine, mainly produced by macrophages and implicated in myofibroblast activation, collagen synthesis, and inhibition of ECM degradation [33]. In the present study, histopathological examination of lung tissues of hDPP4-Tg mice infected with MERS-CoV revealed signs indicative of progressive pulmonary fibrosis and lung damage, including elevated expression of the profibrotic molecules described above (Figs. 5 and 7). Also, although lung damage was still ongoing at 6 dpi, the expression of some ECM components was slightly decreased. These results were presumed to be due to progressive lung damage caused by pulmonary fibrosis.

Recently, macrophages have been reported to be involved in the pathogenesis of tissue fibrosis. Macrophages polarize into classically-activated M1 or alternatively-activated M2 macrophages. M1 macrophages are key effector cells for the elimination of pathogens, virus-infected cells, and malignant cells [42, 43]. More importantly, macrophages are central to wound healing and exacerbate profibrotic processes [44]. M1 macrophages are associated with an anti-fibrotic effect through the release of matrix metallo-proteinases that degrade the ECM. M1 macrophages also produce proinflammatory cytokines and chemokines that indirectly promote the proliferation of myofibroblasts and recruit fibrocytes, leading to scar tissue formation. When the tissue damage persists, M2 macrophages are activated, leading to continuous production of TGF-β and growth factors that promote proliferation of myofibroblasts and fibrocytes, activation of the epithelial–mesenchymal/endothelial–mesenchymal transition, and ECM deposition [45]. We found that macrophages were increased and polarized toward an alternatively activated M2 state in the lungs of MERS-CoV-infected hDPP4-Tg mice compared to non-treated control mice (Fig. 6). However, there was no difference in the expression of M1 macrophage-specific markers between the lungs of MERS-CoV-infected and non-treated hDPP4-Tg mice (data not shown). By contrast, as described above, increased levels of proinflammatory cytokines were observed in the lungs of MERS-CoV-infected hDPP4-Tg mice. These findings likely indicate that the inflammatory response and tissue repair proceed in parallel after MERS-CoV infection and suggest that macrophages might be the major effector cells, resulting in exacerbated lung fibrosis and dysfunction. Therefore, further studies investigating this effect would be valuable in enabling us to identify the pathogenesis of MERS-CoV infection.

In summary, the hDPP4-Tg mice developed in this study were efficiently infected by MERS-CoV to exhibit morbidity and mortality with minimal perturbation caused by overexpressed DPP4. Therefore, these established hDPP4-Tg mice are useful as an animal model for investigating the pathogenesis of MERS-CoV infection and for research into effective preventive and therapeutic agents.

## Figures and Tables

**Table 1:** Sequences of the primers used for qRT-PCR.

Gene	Primer sequences
DPP4	F: 5’-CCA AAG ACT GTA CGG GTT CC-3’
	R: 5’-TCA ACA TAG AAG CAG GAG CAG-3’
IL-1β	F: 5’-TGG ACC TTC CAG GAT GAG GAC A-3’
	R: 5’-GTT CAT CTC GGA GCC TGT AGT G-3’
TNF-α	F: 5’-CAT CTT CTC AAA ATT CGA GTG ACA A-3’
	R: 5’-TGG GAG TAG ACA AGG TAC AAC CC-3’
MIP-1α	F: 5’-ACC ATG ACA CTC TGC AAC C-3’
	R: 5’-CGA TGA ATT GGC GTG GAA TC-3’
CD14	F: 5’-CTC TGT CCT TAA AGC GGC TTA C-3’
	R: 5’-GTT GCG GAG GTT CAA GAT GTT-3’
Ly6C	F: 5’-GCA GTG CTA CGA GTG CTA TGG-3’
	R: 5’-ACT GAC GGG TCT TTA GTT TCC TT-3’
Ym-1	F: 5’-GGG CAT ACC TTT ATC CTG AG-3’
	R: 5’-CCA CTG AAG TCA TCC ATG TC-3’
Fizz-1	F: 5’-CCC TCC ACT GTA ACG AAG ACT C-3’
	R: 5’-CAC ACC CAG TAG CAG TCA TCC-3’
Arginase1	F: 5’-CTC CAA GCC AAA GTC CTT AGA G-3’
	R: 5’-AGG AGC TGT CAT TAG GGA CAT C-3’
Col Iα1	F: 5’-GCT CCT CTT AGG GGC CAC T-3’
	R: 5’- CCA CGT CTC ACC ATT GGG G-3’
Col III	F: 5’-GTT CTA GAG GAT GGC TGT ACT AAA CAC A-3’
	R: 5’-TTG CCT TGC GTG TTT GAT ATT C-3’
Osteonectin	F: 5’-CCA CAC GTT TCT TTG AGA CC-3’
	R: 5’-GAT GTC CTG CTC CTT GAT GC-3’
Osteopontin	F: 5’-TGA TCA GGA CAA CAA CGG AA-3’
	R: 5’-TCT CCT GGC TCT CTT TGG AA-3’
TGF-β1	F: 5’-CCA CTC GCT TCT TTG AGA CC-3’
	R: 5’-TAG TGG AAG TGG GTG GGG AC-3’
β-actin	F: 5’-CGT ACC ACA GGC ATT GTG A-3’
	R: 5’-CTC GTT GCC AAT AGT GAT GA-3’
upE	F: 5’-GCC TCT ACA CGG GAC CCA TA-3’
	R: 5’-GCA ACG CGC GAT TCA GTT-3’

Primers used to measure the expression levels of genes associated with inflammatory and pathological responses. The β-actin gene was used as an endogenous control.

F and R, sequences of the forward and reverse primers, respectively.
